# A Visualized Nomogram for Predicting Prognosis in Elderly Patients after Percutaneous Coronary Intervention

**DOI:** 10.31083/j.rcm2505155

**Published:** 2024-05-06

**Authors:** Qin Chen, Yuxiang Chen, Ruijin Hong, Jiaxin Zhong, Lihua Chen, Yuanming Yan, Lianglong Chen, Yukun Luo

**Affiliations:** ^1^Fujian Key Laboratory of Vascular Aging (Fujian Medical University), Department of Cardiology, Fujian Medical University Union Hospital, 350001 Fuzhou, Fujian, China; ^2^Fujian Key Laboratory of Vascular Aging (Fujian Medical University), Fujian Institute of Coronary Heart Disease, 350001 Fuzhou, Fujian, China; ^3^Fujian Key Laboratory of Vascular Aging (Fujian Medical University), Fujian Heart Medical Center, 350001 Fuzhou, Fujian, China

**Keywords:** nomogram, target vessel revascularization, percutaneous coronary intervention, coronary artery disease, quantitative flow ratio

## Abstract

**Background:**

Revascularized patients still experience adverse cardiovascular events. This is particularly true for elderly patients over the age of 65, as they often have more co-morbid vascular conditions. It is important to develop a tool to assist clinicians in comprehensively assessing these patients’ prognosis. The objective of this study is to create a comprehensive visual nomogram model combining clinical and physiological assessments to predict outcomes in elderly patients undergoing percutaneous coronary intervention (PCI).

**Methods:**

This study is a retrospective investigation of patients who underwent PCI between January 2016 and December 2017. A total of 691 patients with 1461 vessels were randomly divided into a training (n = 483) and a validation set (n = 208). A multivariate Cox regression model was employed using the training set to select variables for constructing a nomogram. The performance of the nomogram was assessed through the receiver operating characteristic curve (ROC) and calibration curves to evaluate its discrimination and predictive accuracy. To further assess the clinical usefulness, Kaplan–Meier curve analysis and landmark analysis were conducted.

**Results:**

Independent risk factors, including diabetes mellitus (DM), post-PCI quantitative flow ratio (QFR), previous myocardial infarction (MI), and previous PCI, were contained in the nomogram. The nomogram exhibited a good area under the curve (AUC) ranging from 0.742 to 0.789 in the training set, 0.783 to 0.837 in the validation set, and 0.764 to 0.786 in the entire population. Calibration curves demonstrated a well-fitted curve in all three sets. The Kaplan–Meier curves showed clear separation and the patients with higher scores in the nomogram model exhibited a higher incidence of target vessel revascularization (TVR) (7.99% vs. 1.24% for 2-year, *p* < 0.001 and 13.54% vs. 2.23% for 5-years, *p* < 0.001, respectively).

**Conclusions:**

This study has developed the visually intuitive nomogram to predict the 2-year and 5-year TVR rates for elderly patients who underwent PCI. This tool provides more accurate and comprehensive healthcare guidance for patients and their physicians.

## 1. Introduction

The presence of myocardial ischemia greatly affects the prognosis of patients with coronary artery disease (CAD), leading to higher mortality and increased complications. Percutaneous coronary intervention (PCI) is widely recognized as an effective approach to improve patient outcomes in patients with CAD. Previous randomized controlled trials (RCTs) have further reinforced the clinical acceptance of PCI as a suitable therapeutic method for managing CAD [1, 2, 3, 4]. However, even after successful revascularization, patients still experience various adverse cardiovascular events, including death, myocardial infarction (MI), target vessel revascularization (TVR), and target vessel failure (TVF) [5, 6, 7].

Elderly adults, specifically those aged 65 years and above, are particularly vulnerable to developing CAD. In a study conducted by Gupta *et al*. [8] and Chen JL *et al*. [9], it was revealed that this age group experiences significantly higher rates of MI and mortality within the CAD population. As a result, elderly patients are recognized as a high risk group, and their management and the prediction of their prognosis is important for not only prolonging their lifespan, but also enhancing their quality of life [10].

Previous research shows that clinical prognosis is influenced by both physiological assessments and systemic factors such as gender, age, race, and diabetes mellitus (DM) [11, 12, 13]. Most studies have primarily focused on predicting prognosis based on either clinical characteristics or coronary artery pathology. However, it is worth considering whether a more effective approach would be to integrate both clinical characteristics and physiological assessments in order to predict clinical prognosis. This approach may provide more accurate and comprehensive predictions for patients with coronary artery disease.

In light of the above, this study aims to develop a prognostic nomogram model to enhance TVR prediction in elderly PCI patients and validate its reliability and utility.

## 2. Materials and Methods

### 2.1 Study Population

A retrospective analysis was conducted on consecutive patients from January 2016 to December 2017 at Fujian Medical University Union Hospital. The study focused on patients who were 65 years or older and underwent PCI, with a 5-year clinical follow-up period. The exclusion criteria for the study were: (1) patients who were under 65 years old, (2) acute myocardial infarction (AMI) within 7 days [14], (3) lack of follow-up data, (4) situations where quantitative flow ratio (QFR) calculation could not be performed, including an interrogated lesion involving a myocardial bridge or bypass graft; severe overlap in the stenosed segment or severe tortuosity of any interrogated vessel; and poor angiographic image quality.

### 2.2 QFR Computation and Quantitative Coronary Angiography (QCA)

The QFR computations and QCA analyses were performed by two independent investigators blinded to the clinical data using the AngioPlus system (Pulse Medical Imaging Technology Shanghai, China) according to standard operating procedures. The three-dimensional (3D) reconstructions of three main vessels were performed based on the automated contouring of two angiographic projections captured at 15 frames/s and at least 25° apart. After 3D reconstruction, QFRs were computed using contrast flow velocity models [15]. In this study, QFR was computed in each participant at the time of pre-PCI and post-PCI. Post-PCI QFR, defined as including at least one lesion treated with PCI, was retrospectively computed in all eligible vessels [16]. The QCA information of all vessels consisted of blood flow resistance (BFR), percent diameter stenosis (DS%) and the percent of area stenosis (AS%). Furthermore, the QFR and QCA data for patients with multiple lesions are presented as the mean of those values.

### 2.3 Data Collection and Follow-up

An electronic medical record system was utilized to retrospectively gather relevant clinical data of the patients at the time of their first hospitalization. This allowed the researchers to access and analyze the necessary information for their analysis. Laboratory indices during the initial hospitalization such as low-density lipoprotein cholesterol (LDL-C), creatinine, N-terminal pro brain natriuretic peptide (NT-proBNP), and high-sensitivity C-reactive protein (hs-CRP) were calculated using standard laboratory techniques. Echocardiography was employed to determine left ventricular ejection fraction (LVEF) and E/E’. E/E’ is the ratio of peak mitral early filling velocity (E) to early diastolic mitral annular velocity (E’), an indicator of diastolic cardiac function. All patients received standard pharmacological treatment according to clinical guidelines, and the information on medications used, including statins, antiplatelet agents, angiotensin-converting-enzyme inhibitors (ACEIs), and angiotensin-receptor blockers (ARBs), was reported. Information of other atherosclerosis-related diseases, such as the atherosclerosis of the carotid arteries and strokes, and in patients with atrial fibrillation and their use of anticoagulants, was also obtained.

Target vessel revascularization (TVR) was defined as any subsequent PCI or surgical bypass involving any segment of the target vessel, including the target lesion and non–target lesions that underwent revascularization. Target vessel and non-target lesion revascularization was defined as any repeat percutaneous intervention or surgical bypass of the target vessel for pre-existing disease, disease progression or other reasons unrelated to the target lesion. Additionally, the definition of target vessel was the entire major intervened coronary vessel, including side branches [17]. We analyzed patients with TVR as our endpoint. Furthermore, TVR was performed through detecting the significant stenosis by angiography based on whether patients suffered from chest pain or other symptoms. And those who required repeat revascularization were also included in the TVR groups.

### 2.4 Statistical Analysis

Continuous variables were presented as mean ± standard deviation (SD) or median (interquartile range, IQR) and compared using appropriate statistical tests such as the 2-sample Student’s *t*-test, Welch’s *t*-test, or Mann-Whitney U test. Categorical variables were presented as numbers and percentages and compared using the chi-squared test or Fisher’s exact test.

To develop the overall survival nomogram, a multivariable Cox regression model was chosen in a training set. Initially, univariable Cox regression was performed, and variables with a *p*-value < 0.10 were selected as candidate variables. The nomogram’s performance was assessed through discrimination and calibration in both the training set and the validation set. The receiver operating characteristic curve (ROC) was used to measure the area under the curve (AUC). The optimal threshold was determined using the Youden index derived from the ROC curve. Model performance was further evaluated through survival analysis using Kaplan–Meier curves and landmark analysis. Calibration plots were used to compare the actual Kaplan–Meier survival estimates with the predicted probability of freedom from 2-year and 5-year TVR, and C-index was used to assess the performance of the nomogram.

In this study, the death is a competing risk data, which affect the incidence of TVR during follow-up. Fine-Gray model was performed to analysis this phenomenon. We categorized the population into three groups: the deceased individuals as the competitive risk subgroup, those experiencing TVR as the risk group, and the rest of the population as the control group. Subsequently, we performed Fine-Gray analysis over a 5-year follow-up, plotted the curves, and meticulously observed the outcomes.

All statistical analyses were performed using SPSS 25.0 (IBM Inc., New York, NY, USA) and R version 4.2.2 (R Foundation for Statistical Computing, Vienna, Austria).

## 3. Results

### 3.1 Study Population, Baseline Characteristics and Outcomes

**Fig. 1.**
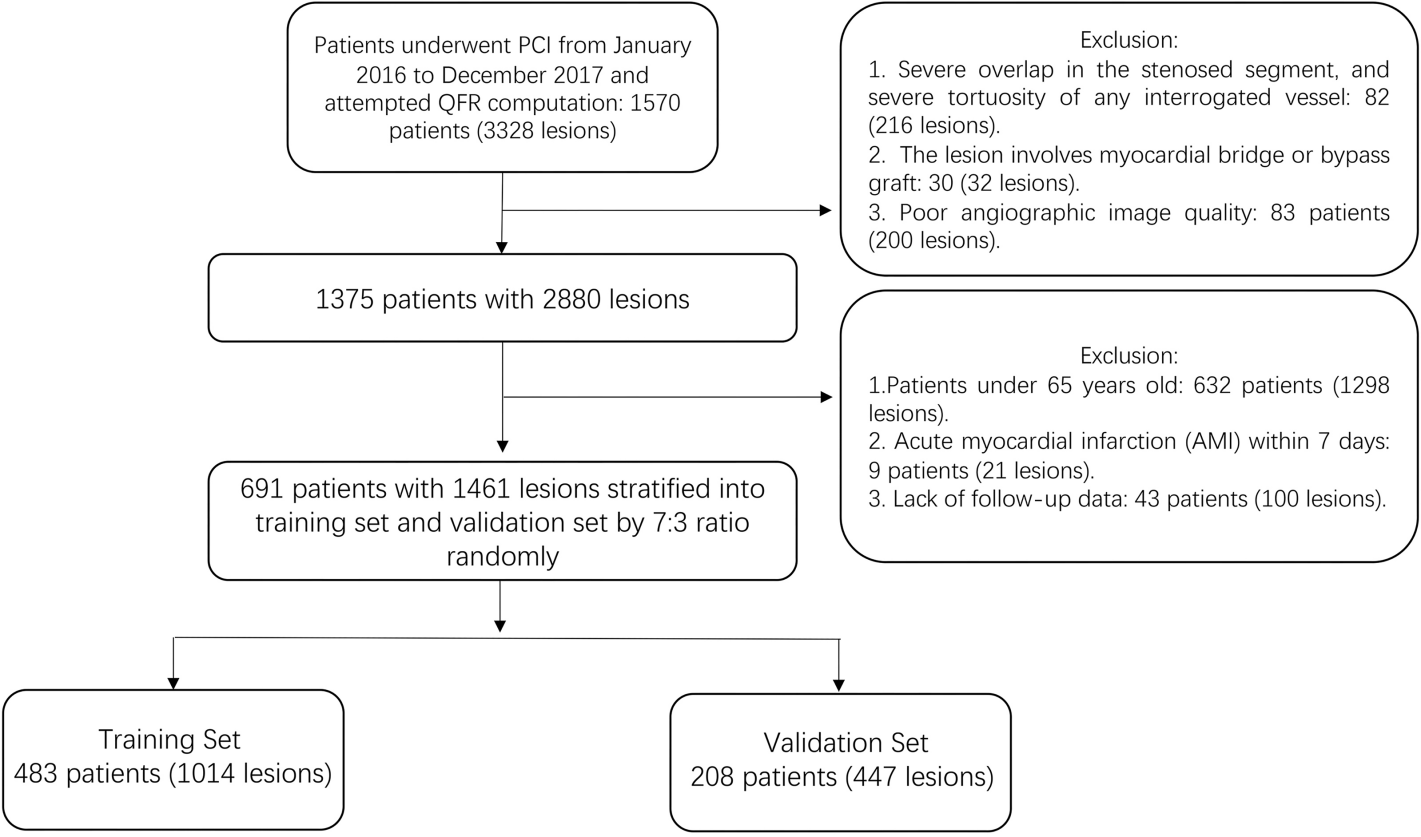
**Study flow chart**. PCI, percutaneous coronary intervention; QFR, quantitative flow ratio.

From January 2016 to December 2017, the angiographic data of 3328 vessels from 1570 patients were screened. A total of 448 vessels in 195 patients were excluded due to a failure in computing QFR, 1419 vessels in 684 patients were excluded due to unsuitable clinical conditions, resulting in 1461 vessels from 691 patients being included in the final analysis. The enrolled patients were randomly stratified into a training set and a validation set at a 7:3 ratio. Finally, 483 patients with 1014 lesions were divided into a training set, and 208 patients with 447 lesions were divided into a validation set (Fig. 1).

Tables 1,2 provided baseline characteristics and angiographic data of the training set and validation set. There were no statistically significant differences in the general clinical and angiographic data between both sets. During the follow-up period of 2 and 5 years, the mean of the overall observation period was 56 months. Additionally, 61 patients died during following up, and the remaining population had an average follow-up period of 58.3 months. A total of 28 patients during 2 years and 48 patients within 5 years experienced TVR. The rates of TVR were 4.05% and 6.95%, respectively. In the training set, the TVR rates after 2 years and 5 years were 4.55% and 7.66% respectively. Similarly, in the validation set, the TVR rates after 2 years and 5 years were 2.88% and 5.29% respectively. No significant differences were observed between the two groups in terms of TVR rates (all *p* > 0.05).

**Table 1.** **Clinical characteristics**.

Variable		Overall	Training set	Validation set	*p*-value
		(N = 691)	(N = 483)	(N = 208)	
Study population					
	Age, year	73.00 ± 5.88	73.10 ± 5.99	72.77 ± 5.63	0.655
	Male	490 (70.91%)	349 (72.26%)	141 (67.79%)	0.236
Clinical presentation					
	Unstable angina	405 (58.61%)	279 (57.76%)	126 (60.58%)	0.491
	STEMI	95 (13.75%)	67 (13.87%)	28 (13.46%)	0.886
	NSTEMI	130 (18.81%)	95 (19.67%)	35 (16.83%)	0.381
	Stable angina	60 (8.68%)	41 (8.49%)	19 (9.13%)	0.782
	Atrial fibrillation	22 (3.18%)	15 (3.11%)	7 (3.37%)	0.676
	Strokes	33 (4.78%)	23 (4.76%)	10 (4.81%)	0.889
	Other atherosclerosis diseases	13 (1.88%)	9 (1.86%)	4 (1.92%)	0.279
Cardiovascular risk factor					
	Hypertension	523 (75.69%)	362 (74.94%)	161 (77.40%)	0.490
	Diabetes mellitus	264 (38.21%)	188 (38.92%)	76 (36.53%)	0.554
	Current smoker	301 (43.56%)	208 (43.06%)	93 (44.71%)	0.689
	Previous MI	61 (8.83%)	38 (7.87%)	23 (11.06%)	0.175
	Previous PCI	85 (12.30%)	58 (12.01%)	27 (12.98%)	0.721
Laboratory indices					
	NT-proBNP, pg/mL	1561.24 ± 4199.68	1488.20 ± 3884.60	1732.43 ± 4865.96	0.630
	Hs-CRP, mg/L	12.28 ± 26.88	11.80 ± 25.95	13.39 ± 28.98	0.695
	Cr, umol/L	91.45 ± 67.40	89.42 ± 61.76	96.16 ± 78.93	0.107
	LDL-C, umol/L	2.76 ± 0.98	2.77 ± 0.99	2.72 ± 0.97	0.497
	LVEF, %	59.63 ± 11.81	59.29 ± 12.07	60.40 ± 11.19	0.335
	E/e’	14.51 ± 5.86	14.51 ± 5.91	14.50 ± 5.76	0.966
Medication					
	Statin	680 (98.41%)	475 (98.34%)	205 (98.56%)	0.999
	Antiplatelet	690 (99.86%)	482 (99.79%)	208 (100%)	0.999
	ACEIs or ARBs	460 (66.57%)	325 (67.29%)	135 (64.90%)	0.236
	Anticoagulants	20 (2.89%)	14 (2.90%)	6 (2.88%)	0.776
TVR rates					
	2-year	28 (4.05%)	22 (4.55%)	6 (2.88%)	0.307
	5-year	48 (6.95%)	37 (7.66%)	11 (5.29%)	0.261

Values are mean ± SD, n (%) or median (interquartile range). MI, myocardial infarction; PCI, percutaneous coronary intervention; STEMI, ST-segment elevation myocardial infarction; NSTEMI, non-ST segment elevation myocardial infarction; ACEIs or ARBs, angiotensin-converting-enzyme inhibitors or angiotensin-receptor blockers; LDL-C, low-density lipoprotein cholesterol; NT-proBNP, N-terminal pro brain natriuretic peptide; Hs-CRP, high-sensitivity C-reactive protein; LVEF, left ventricular ejection fraction; Cr, creatinine; TVR, target vessel revascularization; SD, standard deviation; E/e’, ratio of early diastolic mitral flow velocity to early diastolic mitral ring motion velocity.

**Table 2.** **Angiographic characteristics and QFR analysis**.

Variable		Overall	Training set	Validation set	*p*-value
		(Nv = 1461)	(Nv = 1014)	(Nv = 447)	
Pre-PCI					
	LAD	568 (38.88%)	394 (38.86%)	174 (38.93%)	
	LCX	428 (29.30%)	301 (29.68%)	127 (28.41%)	
	RCA	465 (31.83%)	319 (31.46%)	146 (32.66%)	
	AS%	48.05 ± 16.23	47.78 ± 16.18	48.66 ± 16.35	0.449
	DS%	67.39 ± 17.53	67.05 ± 17.78	68.17 ± 16.93	0.443
	QFR	0.77 ± 0.18	0.77 ± 0.18	0.76 ± 0.19	0.260
	BFR, mmHg×s/m	187.46 ± 112.71	186.54 ± 112.64	189.55 ± 112.98	0.596
Post-PCI					
	AS%	15.70 ± 15.38	16.01 ± 15.52	15.00 ± 15.08	0.272
	DS%	23.70 ± 23.30	24.17 ± 23.45	22.64 ± 22.96	0.215
	QFR	0.96 ± 0.06	0.95 ± 0.07	0.96 ± 0.06	0.745
	BFR, mmHg×s/m	124.66 ± 127.11	123.41 ± 126.84	127.66 ± 127.96	0.384

Values are mean ± SD. PCI, percutaneous coronary intervention; DS%, diameter stenosis percentage; AS%, area stenosis percentage; BFR, blood flow resistance; QFR, quantitative flow ratio; LAD, left anterior descending artery; LCX, left circumflex artery; RCA, right coronary artery; Nv, number of vessels; SD, standard deviation.

### 3.2 Development of the Multivariate Prognostic Nomogram to Predict TVR

Appropriate variables from patients in the training set were selected to develop the nomogram model. The clinical characteristics and angiographic data of the training set are shown in Tables 3,4. Patients in the training set were further stratified into a control group and a TVR group. Patients in the TVR group experienced higher rates of DM, previous MI, and previous PCI compared to the control group (56.76% vs. 37.44%, *p* = 0.021; 32.43% vs. 5.83%, *p* < 0.001; 40.54% vs. 9.64%, *p* < 0.001). There were no significant differences in the QCA analysis in the pre-PCI (all *p* > 0.05). The conditions of the vessels after PCI were similar but the QFR of the TVR group was less than the control group (0.93 ± 0.08 vs. 0.96 ± 0.06, *p* = 0.001).

**Table 3.** **Clinical characteristics for training set**.

Variable		Overall	Control group	TVR group	*p*-value
		(N = 483)	(N = 446)	(N = 37)	
Study population					
	Age, year	73.10 ± 5.99	73.10 ± 6.04	73.00 ± 5.47	0.858
	Male	349 (72.26%)	320 (71.75%)	29 (78.38%)	0.387
Clinical presentation					
	Diabetes mellitus	188 (38.92%)	167 (37.44%)	21 (56.76%)	0.021
	Hypertension	362 (74.94%)	333 (74.66%)	29 (78.38%)	0.616
	Current smoker	208 (43.06%)	188 (42.15%)	20 (54.05%)	0.160
	Previous MI	38 (7.87%)	26 (5.83%)	12 (32.43%)	<0.001
	Previous PCI	58 (12.01%)	43 (9.64%)	15 (40.54%)	<0.001
Cardiovascular risk factor					
	Unstable angina	279 (57.76%)	261 (58.52%)	18 (48.65%)	0.243
	STEMI	67 (13.87%)	60 (13.45%)	7 (18.91%)	0.355
	NSTEMI	95 (19.67%)	90 (20.18%)	5 (13.51%)	0.327
	Stable angina	41 (8.49%)	34 (7.62%)	7 (18.92%)	0.028
	Atrial fibrillation	15 (3.11%)	14 (3.14%)	1 (2.70%)	0.279
	Strokes	23 (4.76%)	21 (4.71%)	2 (5.41%)	0.146
	Other atherosclerosis diseases	9 (1.86%)	8 (1.79%)	1 (2.70%)	0.221
Laboratory indices					
	NT-proBNP, pg/mL	1488.20 ± 3884.60	1473.67 ± 3671.50	1663.00 ± 5951.61	0.980
	Hs-CRP, mg/L	11.80 ± 25.95	12.39 ± 26.90	4.88 ± 5.53	0.184
	Cr, umol/L	89.42 ± 61.76	88.46 ± 55.43	101.01 ± 113.75	0.795
	LDL-C, umol/L	2.77 ± 0.99	2.78 ± 0.98	2.66 ± 1.02	0.351
	LVEF, %	59.29 ± 12.07	59.60 ± 12.01	55.61 ± 12.35	0.057
	E/e’	14.51 ± 5.91	14.47 ± 5.94	14.91 ± 5.63	0.660
Medication					
	Statin	475 (98.34%)	438 (98.21%)	37 (100%)	0.999
	Antiplatelet	482 (99.79%)	445 (99.78%)	37 (100%)	0.999
	ACEIs or ARBs	325 (67.29%)	300 (67.26%)	25 (67.57%)	0.970
	Anticoagulants	14 (2.90%)	13 (2.91%)	1 (2.70%)	0.992

Values are mean ± SD or n (%) or median (interquartile range). MI, myocardial infarction; PCI, percutaneous coronary intervention; STEMI, ST-segment elevation myocardial infarction; NSTEMI, non-ST segment elevation myocardial infarction; ACEIs or ARBs, angiotensin-converting-enzyme inhibitors or angiotensin-receptor blockers; LDL-C, low-density lipoprotein cholesterol; NT-proBNP, N-terminal pro brain natriuretic peptide; Hs-CRP, high-sensitivity C-reactive protein; LVEF, left ventricular ejection fraction; Cr, creatinine; TVR, target vessel revascularization; SD, standard deviation; E/e’, ratio of early diastolic mitral flow velocity to early diastolic mitral ring motion velocity.

**Table 4.** **Angiographic characteristics and QFR analysis for training set**.

Variable		Overall	Control group	TVR group	*p*-value
		(Nv = 1014)	(Nv = 931)	(Nv = 83)	
Pre-PCI					
	LAD	394 (38.86%)	364 (39.10%)	30 (36.14%)	
	LCX	301 (29.68%)	275 (29.53%)	26 (31.33%)	
	RCA	319 (31.46%)	292 (31.36%)	27 (32.53%)	
	AS%	47.78 ± 16.18	48.00 ± 16.12	45.30 ± 16.79	0.240
	DS%	67.05 ± 17.78	67.34 ± 17.61	63.76 ± 19.45	0.128
	QFR	0.77 ± 0.18	0.77 ± 0.18	0.78 ± 0.18	0.744
	BFR, mmHg×s/m	186.54 ± 112.64	186.07 ± 113.16	191.79 ± 107.16	0.492
Post-PCI					
	AS%	16.01 ± 15.52	15.73 ± 15.38	19.20 ± 16.74	0.067
	DS%	24.17 ± 23.45	23.68 ± 23.22	29.57 ± 25.38	0.055
	QFR	0.95 ± 0.07	0.96 ± 0.06	0.93 ± 0.08	0.001
	BFR, mmHg×s/m	123.41 ± 126.84	121.54 ± 127.28	142.88 ± 121.70	0.163

Values are mean ± SD. PCI, percutaneous coronary intervention; DS%, diameter stenosis percentage; AS%, area stenosis percentage; BFR, blood flow resistance; QFR, quantitative flow ratio; LAD, left anterior descending artery; LCX, left circumflex artery; RCA, Right coronary artery; TVR, target vessel revascularization; Nv, number of vessels; SD, standard deviation.

**Fig. 2.**
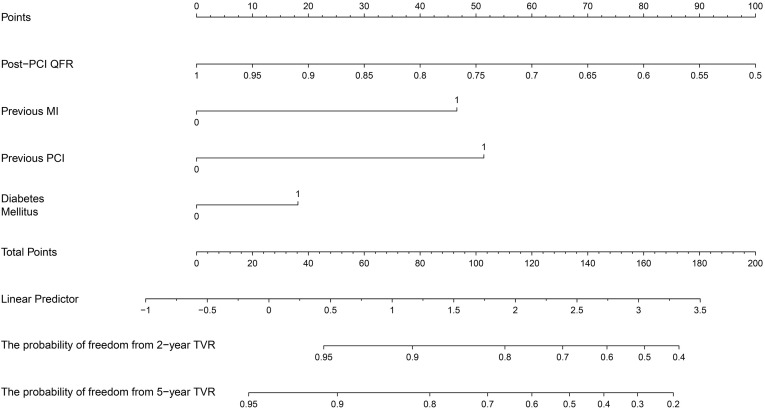
**Nomogram for predicting 2-year and 5-year TVR**. Points were composed of diabetes mellitus, post-PCI QFR, previous MI and previous PCI. The score for each value was assigned by drawing a line upward to the points line, and the sum of the four scores was plotted on the total points line. Finally, the probability line was used to determine the probability of freedom from 2-year and 5-year TVR. MI, myocardial infarction; QFR, quantitative flow ratio; TVR, target vessel revascularization; PCI, percutaneous coronary intervention.

According to the above baseline characteristics, angiographic data and risk factors of cardiovascular hazard events, 13 variables were selected into the univariate Cox regression analysis (Table 5). 5 candidate variables were found to satisfy the threshold of *p* < 0.10. The multivariate Cox regression analysis indicated that DM, previous MI, previous PCI, and post-PCI QFR were significant independent predictors of the rate of TVR in the training set (*p* < 0.05). We eventually created a nomogram for TVR prediction by using these factors (Fig. 2). Each predictor corresponded to a specific point by drawing a straight line upwards to the axis point. Scores for each variable were added and located on the “Total Points” axis. Finally, a vertical line was drawn straight down from the plotted total axis point to the 2-year or 5-year TVR probability axis to determine the probability of TVR. The Nomogram score was calculated using the following formula:

Nomogram score = [200 + (–200 × post-PCI QFR)] + (51.41004 × previous PCI) + (46.5815 × previous MI) + (18.14027 × DM)

**Table 5.** **Independent predictors of 5-year TVR**.

	Univariate model			Multivariate model		
	HR	95% CI	*p*-value	HR	95% CI	*p*-value
Age	0.99	0.95–1.06	0.982			
Male	1.43	0.65–3.13	0.371			
Current smoker	1.60	0.84–3.04	0.157			
Stable angina	2.26	1.12–5.83	0.025	1.18	0.46–2.43	0.717
Previous MI	6.43	3.23–12.81	<0.001	3.13	1.34–7.33	0.009
Previous PCI	5.41	2.81–10.44	<0.001	3.47	1.54–7.81	0.003
Diabetes mellitus	2.12	1.11–4.07	0.023	1.85	1.04–3.29	0.038
Hypertension	1.24	0.57–2.71	0.591			
Post-PCI QFR	0.01	0.01–0.35	0.010	0.01	0.01–0.53	0.021
Atrial fibrillation	1.33	0.89–1.77	0.125			
Strokes	1.47	0.88–2.79	0.323			
Other atherosclerosis diseases	1.17	0.44–1.91	0.119			
Anticoagulants	0.88	0.76–1.10	0.203			

MI, myocardial infarction; PCI, percutaneous coronary intervention; QFR, quantitative flow ratio; TVR, target vessel revascularization; HR, hazard ratio; CI, confidence interval.

### 3.3 Assessment and Validation of the Nomogram’s Performance

**Fig. 3.**
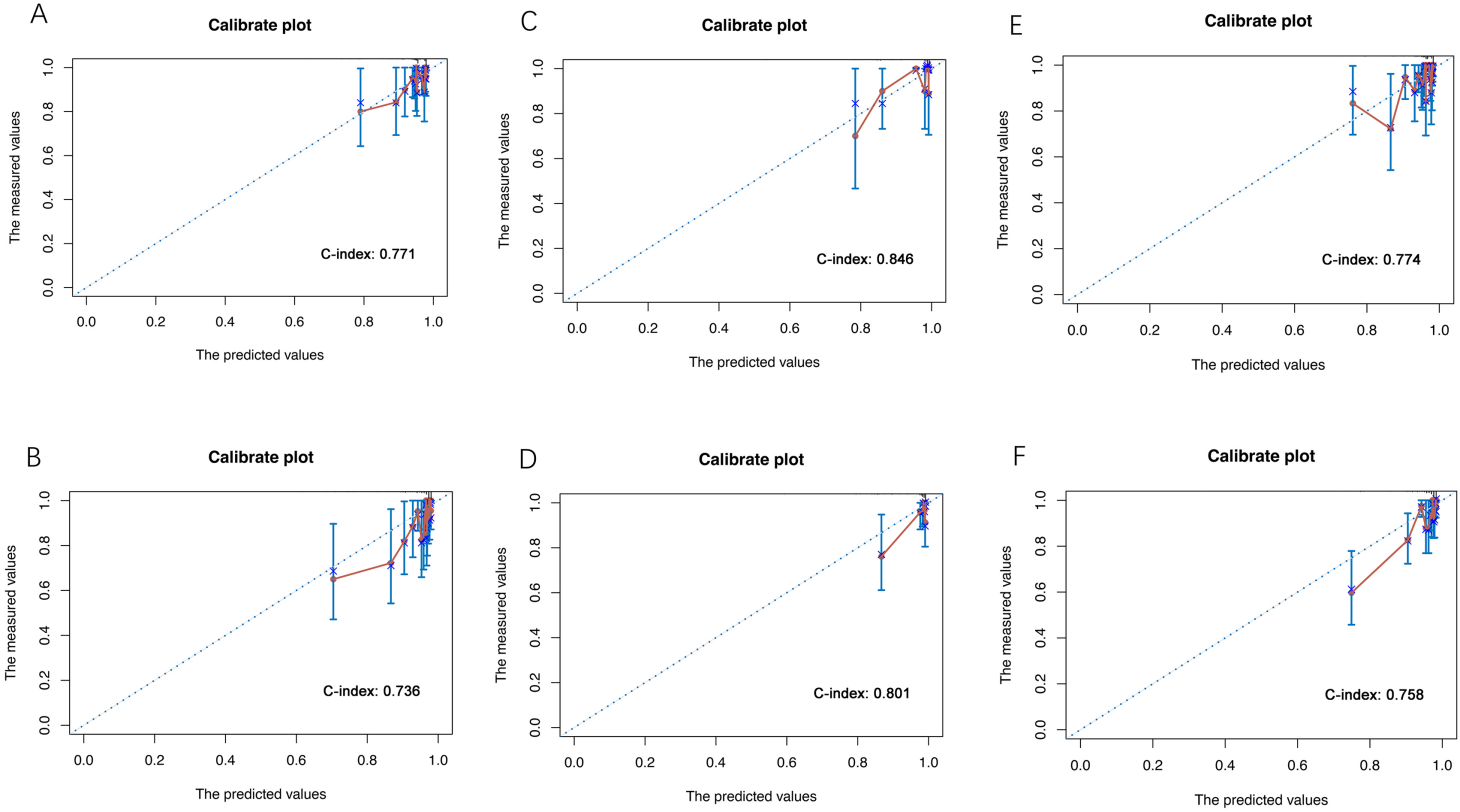
**The calibration plots for predicting 2-year and 5-year TVR probability**. (A) 2-year TVR in the training set. (B) 5-year TVR in the training set. (C) 2-year TVR in the validation set. (D) 5-year TVR in the validation set. (E) 2-year TVR in the whole population. (F) 5-year TVR in the whole population. TVR, target vessel revascularization.

The calibration plots of predictions from the nomogram model in the training set, validation set and total population are displayed in Fig. 3. The C-index was used to assess the performance of the nomogram model. The results revealed that the C-index were 0.771 and 0.736 in the training set during the 2-year and 5-year periods (Fig. 3A,B), 0.846 and 0.801 in the validation set (Fig. 3C,D), 0.774 and 0.758 in the total population (Fig. 3E,F), respectively.

**Fig. 4.**
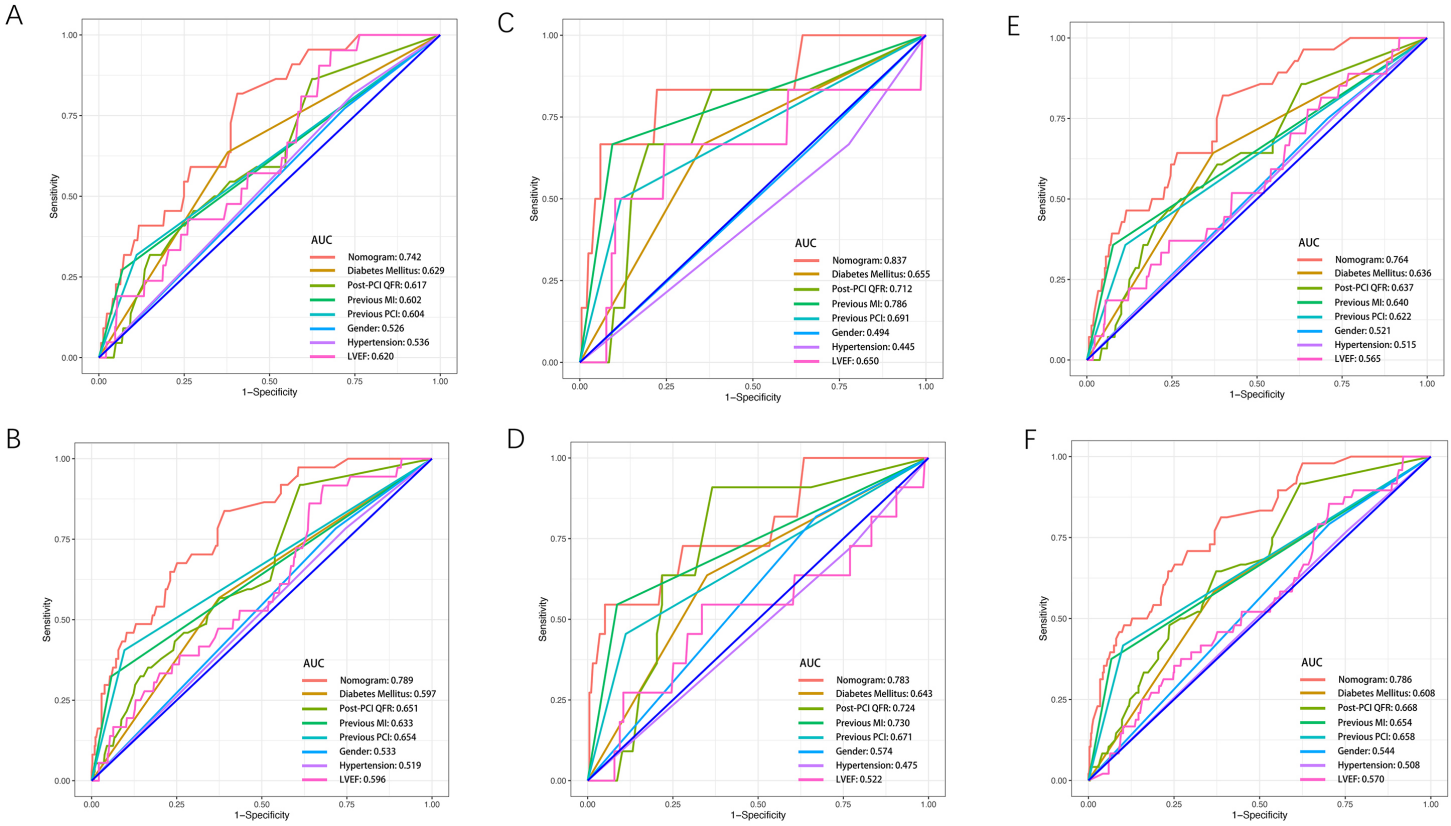
**The ROC curves for comparisons of different variable**. (A) The ROC curves to predict 2-year TVR in training set. (B) The ROC curves to predict 5-year TVR in training set. (C) The ROC curves to predict 2-year TVR in validation set. (D) The ROC curves to predict 5-year TVR in validation set. (E) The ROC curves to predict 2-year TVR in whole population. (F) The ROC curves to predict 5-year TVR in the whole population. ROC, receiver operating characteristic curve; AUC, area under curve; MI, myocardial infarction; QFR, quantitative flow ratio; LVEF, left ventricular ejection fraction; TVR, target vessel revascularization; PCI, percutaneous coronary intervention.

The ROC analysis indicates that the nomogram had an excellent performance for predicting TVR in the training, validation and whole population sets (Fig. 4). The nomogram yielded an AUC of 0.742 and 0.789 for predicting 2-year to 5-year TVR risk in the training set (Fig. 4A,B) and 0.837 and 0.783 in the validation set (Fig. 4C,D). Additionally, the AUC of the nomogram in the total population for predicting 2-year and 5-year TVR were 0.764 and 0.786, respectively (Fig. 4E,F).

**Fig. 5.**
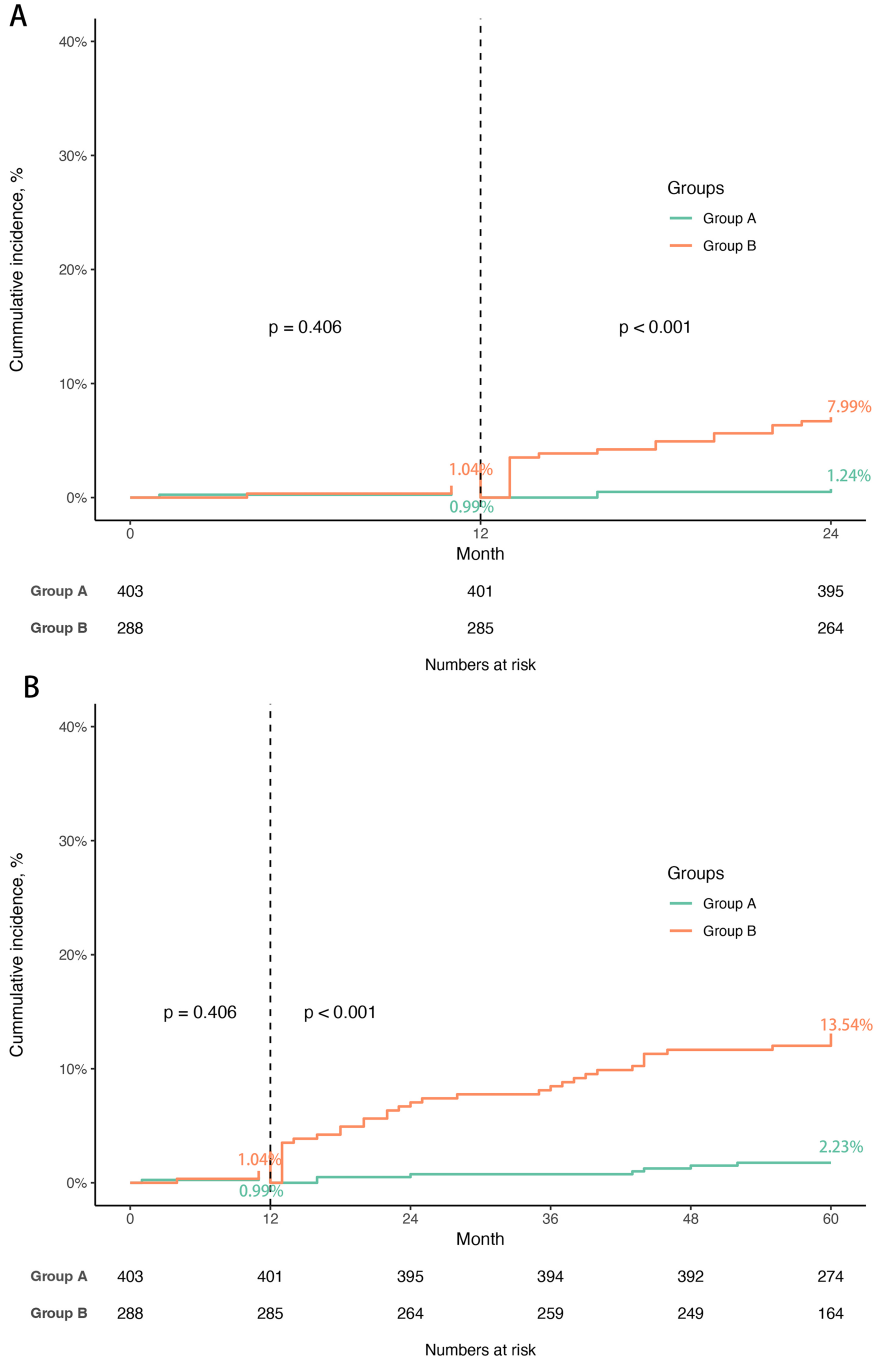
**The Kaplan–Meier curves and landmark analysis to predict TVR**. (A) The Kaplan–Meier curves to predict 2-year TVR and landmark analysis at one year. (B) The Kaplan–Meier curves to predict 5-year TVR and landmark analysis at one year. Group A: score ≤20.07; Group B; score >20.07. The cut-off points of 2-year and 5-year ROC are 20.07. ROC, receiver operating characteristic curve; TVR, target vessel revascularization.

The distribution of the nomogram score in three sets was shown in **Supplementary Fig. 1**. Based on the 2-year and 5-year ROC analysis, a prognostic score cut-off point of 20.07 was determined. The patients were then divided into two groups: Group A (score ≤20.07) and Group B (score >20.07). Kaplan–Meier curves were recorded and a landmark analysis was performed and implemented at 1 year (Fig. 5A,B). The results showed there was no significant difference between the two groups in the incidence of TVR within the first year (log-rank *p* = 0.406) and the curve appeared to be well separated after one year, implying reasonable discrimination (log-rank *p* < 0.001).

### 3.4 Analysis of Competing Risks between Death and TVR

During the follow-up period, we encountered competing risks between the occurrence of death and our primary endpoint, TVR. To further investigate and analyze this situation, we employed the Fine-Gray model, as depicted in **Supplementary Fig. 2**. The results revealed no significant difference in the percentage of death between the groups with scores ≤20.07 and scores >20.07. The incidence of TVR remained higher in the groups with scores >20.07 compared to those with scores ≤20.07 even after accounting for competing risks (*p* < 0.001).

## 4. Discussion

In our cohort of 691 patients aged 65 or older, encompassing a total of 1461 lesions, we have successfully developed and validated a nomogram model. This model more accurately predicts the risk of TVR at 2 years and 5 years. To the best of our knowledge, this is a risk prediction model that integrates both clinical characteristics and physiological assessment values for patients. By incorporating these factors, our model has the potential to serve as a valuable and objective tool, offering insights into the overall condition of patients in clinical practice.

The nomogram model has been utilized extensively in predicting the risk of adverse cardiovascular events [18, 19, 20]. In our research, TVR is acknowledged as an endpoint that has a negative impact on patient prognosis. Among patients who underwent PCI, the occurrence of restenosis or stent thrombosis can adversely affect the long-term patency of the affected blood vessels. Consequently, this necessitates target lesion revascularization or TVR. Furthermore, it is important to note that patients who experience TVR are at a heightened risk for reinfarction and stent thrombosis, further underscoring the importance of effectively predicting and managing this outcome [21].

To evaluate the reliability of the nomogram model, we conducted discrimination and calibration analyses in both the training and validation sets. The calibration plots exhibited favorable calibration across the training set, validation set, and the entire population. The histogram of the nomogram scores showed a concentration of lower scores, and the majority of cases in the calibration plots had predicted values falling within the range of 0.7–1.0. We speculate that this observation can be attributed to the fact that the patients included in this study have undergone PCI, resulting in an overall improvement in their vascular function to some extent. As a result, most cases exhibit relatively low risk scores.

Moreover, the AUC for predicting the risk of TVR at 2 years and 5 years consistently exceeded 0.70 and approached 0.80, indicating good discriminative ability.

However, when comparing the calibration plots and the ROC curves between the 2-year and 5-year predictions, the results for predicting 5-year TVR risk were weaker. This suggests that the model may underestimate the risk of TVR over a longer time period. It is possible that the angiographic results and clinical parameters after PCI are similar and suffer slight changes in the short-term, but the indices of patients may vary as time goes on, leading to the observed differences in prediction accuracy.

In this study, the four most important factors—DM, post-PCI QFR, previous MI and previous PCI—contained the greatest prognostic value and were selected into the nomogram model.

DM is a significant risk factor for cardiovascular diseases, contributing to the development of endothelial dysfunction, vascular inflammation, arterial remodeling, and atherosclerosis. Furthermore, DM is associated with a higher burden of atherosclerosis and is linked to inferior outcomes in patients with CAD [22, 23]. Previous studies have consistently shown that individuals with preexisting diabetes are more prone to experiencing worse outcomes and higher rates of comorbidities. Furthermore, they often display a more severe cardiovascular risk profile [24, 25]. It is crucial to effectively manage and control diabetes in order to mitigate the risk of cardiovascular diseases.

QFR is a novel method to assess vascular physiology with the advantage of omitting extra invasive procedures, and is faster, more efficient, and cost-effective compared to fractional flow reserve (FFR) [26]. Previous studies confirmed the feasibility of QFR calculation in clinical practice, and showed that it was related to higher revascularization and worse adverse cardiac events [27, 28, 29]. Furthermore, Pijls *et al*. [6] concluded that negative correlations were found between post-PCI FFR values and adverse clinical events, and post-PCI FFR was the most significant independent predictor of clinical events. A PANDA III trial revealed that a higher post-PCI QFR was correlated with a better short-term prognosis [16]. Furthermore, according to nomogram model and cut-off value, we recommend aiming for a post-PCI QFR value above 0.9, in addition to considering other risk factors. By doing so, the patient’s score will be minimized, leading to a lower rate of target vessel revascularization events.

A history of prior MI is another predictor in the current study. Zaman *et al*. [30] concluded that patients with a history of CAD are at higher risk despite normal myocardial perfusion. Numerous studies have shown that patients with prior MI experienced a higher risk of MACE or cardiovascular events and history of CAD may be especially important for risk stratification [31, 32, 33]. In this study, previous MI was a stronger risk factor to predict TVR, consistent with previous investigations.

Patients with previous PCI are also at high risk for multiple types of coronary events [34]. A study revealed that an increase in the rates of older and male in the prevalence of atherosclerotic risk factors was found in patients undergoing PCI, and the percentage of revascularization for MI were higher, which may due to the progression of *de novo* lesions at other locations [35, 36]. Thus, in our study, previous PCI was also a notable risk factor for adverse outcomes and was therefore chosen to be included into the model.

The ROC curve was utilized to determine the optimal prognostic score cut-off value. A prognostic score of ≤20.07 was classified as low risk, while a score >20.07 was classified as high risk. Kaplan–Meier curves and landmark analysis demonstrated that the rates of TVR within one year were similar in both groups. This may be attributed to the fact that successfully revascularized vessels tend to exhibit a similar short-term vascular profile, as indicated in Table 2. It is likely that the adverse effects take time to accumulate, resulting in the gradual separation of the curves after one year.

In this study, death was regarded as a competing risk for TVR. To evaluate whether this factor influences the model’s accuracy, we employed the Fine-Gray model. The results indicated that the performance of the nomogram was not impacted by the incidence of death. These findings suggest that the nomogram model possesses excellent clinical practicality.

This model provides a visual representation of risk and is suitable for managing one’s own health proactively. Elderly patients, who are more vulnerable in terms of vascular conditions and overall health, should pay extra attention to their health, effectively control underlying diseases, and undergo regular check-ups if their nomogram score is higher.

### Limitations

Our study has several limitations. First, the present study is a retrospective, single-center analysis, with small a sample size. Therefore, the findings need to be confirmed by further prospective multicenter cohort studies. Data from other centers are also required to access the current model in more external validation sets. Second, not all screened patients were included in the final analysis, which inevitably introduces selection bias. Finally, model performance is not extremely perfect, the prediction of this nomogram is properly performed in lower- risk patients in most cases, and there is room for improvement.

## 5. Conclusions

This study has developed a prognostic nomogram model that incorporates physiological assessment values and three clinical variables: post-PCI QFR, DM, previous MI, and previous PCI. This model provides clinicians with a visualized approach to assess the risk of TVR over a period of two and five years in elderly patients. Moreover, it offers patients more objective and comprehensive health guidance.

## Data Availability

All data generated or analyzed during the current study are included in this article.

## Supplementary Material

Supplementary material associated with this article can be found, in the online version, at https://doi.org/10.31083/j.rcm2505155.
