# Supplementary figures and images for: A Visualized Nomogram for Predicting Prognosis in Elderly Patients after Percutaneous Coronary Intervention
Source: Rev Cardiovasc Med. 2024 May 6;25(5):155. doi: 10.31083/j.rcm2505155 (PMC11267199; doi:10.31083/j.rcm2505155)

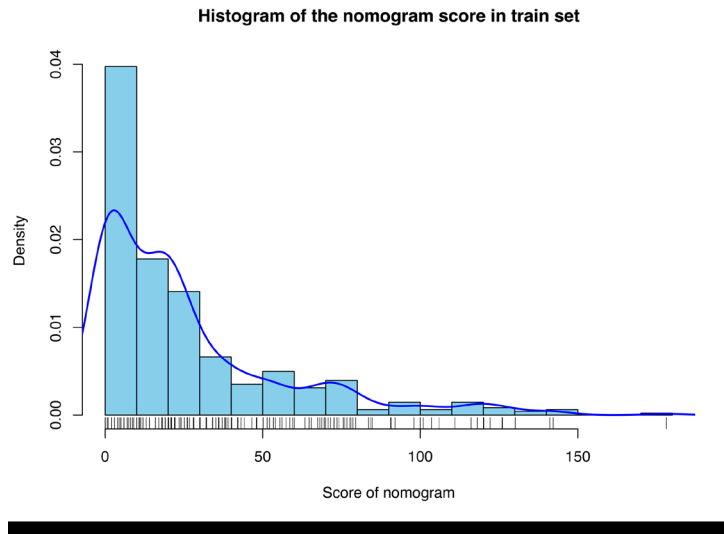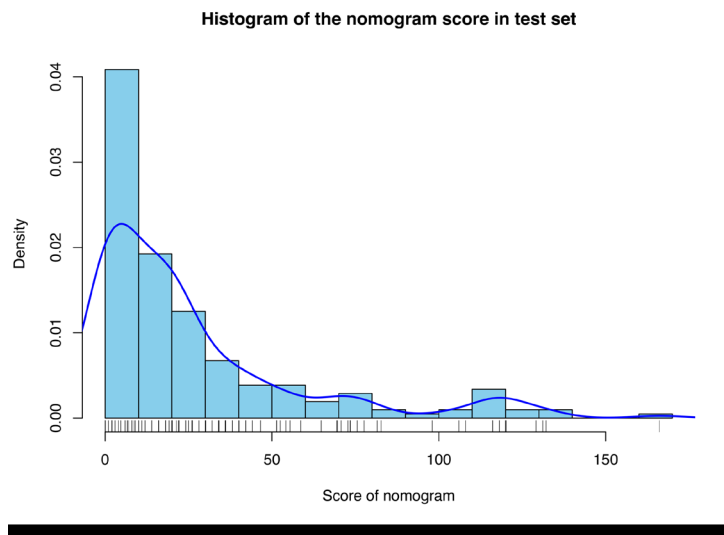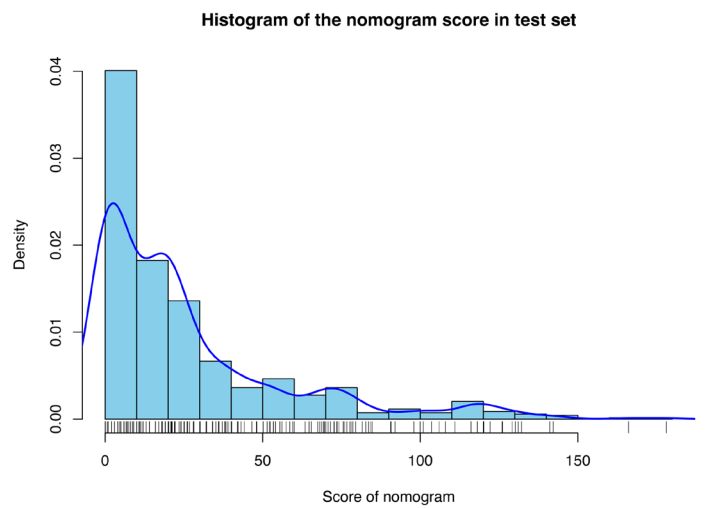

Supplementary Fig. 1

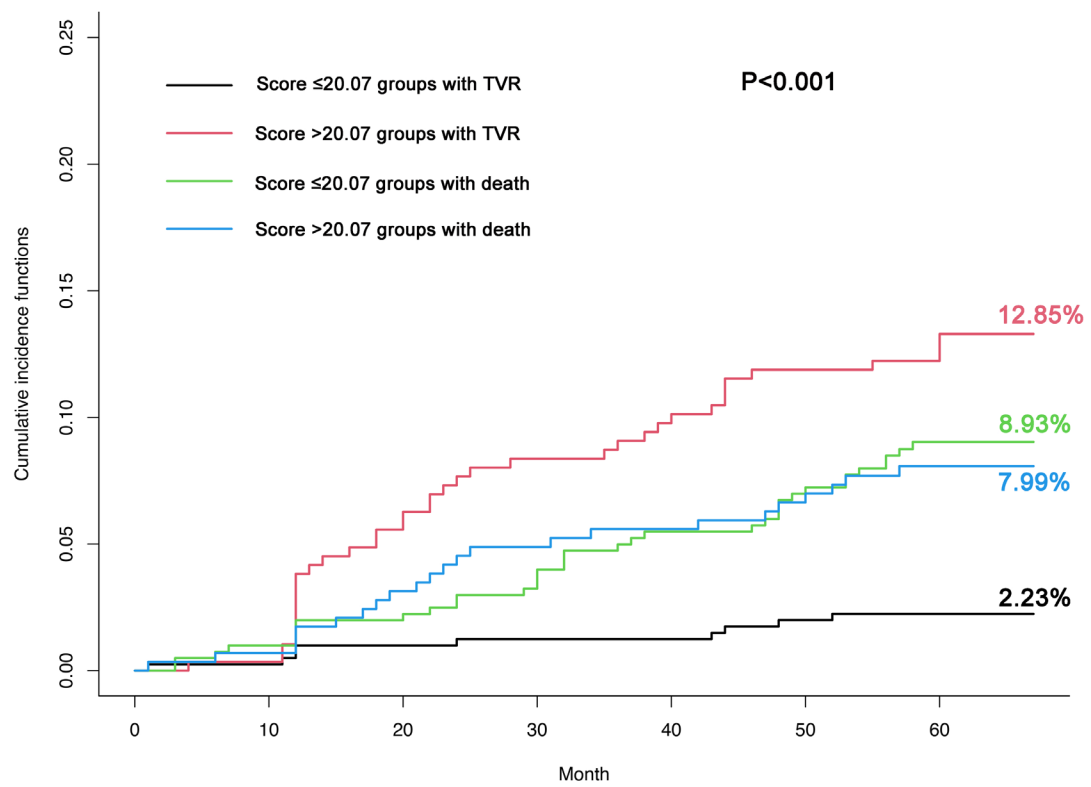

Supplementary Fig. 2

Supplement: Supplementary file 1 [file 2153-8174-25-5-155-s1.pdf]
